# Endogenous antibody responses in REGN-COV2-treated SARS-CoV-2-infected individuals

**DOI:** 10.1093/oxfimm/iqac012

**Published:** 2023-01-06

**Authors:** Ashwini Kurshan, Luke B Snell, Lucie Prior, Jerry C H Tam, Carl Graham, Rajeni Thangarajah, Jonathan D Edgeworth, Gaia Nebbia, Katie J Doores

**Affiliations:** Department of Infectious Diseases, School of Immunology & Microbial Sciences, King’s College London, London, UK; Department of Infectious Diseases, School of Immunology & Microbial Sciences, King’s College London, London, UK; Centre for Clinical Infection and Diagnostics Research, Department of Infectious Diseases, Guy’s and St Thomas’ NHS Foundation Trust, London, UK; Department of Infectious Diseases, School of Immunology & Microbial Sciences, King’s College London, London, UK; Department of Infectious Diseases, School of Immunology & Microbial Sciences, King’s College London, London, UK; Department of Infectious Diseases, School of Immunology & Microbial Sciences, King’s College London, London, UK; Department of Infectious Diseases, Guy’s and St Thomas’ NHS Foundation Trust, London, UK; Department of Pharmacy, Guy’s and St Thomas’ NHS Foundation Trust, London, UK; Centre for Clinical Infection and Diagnostics Research, Department of Infectious Diseases, Guy’s and St Thomas’ NHS Foundation Trust, London, UK; Centre for Clinical Infection and Diagnostics Research, Department of Infectious Diseases, Guy’s and St Thomas’ NHS Foundation Trust, London, UK; Department of Infectious Diseases, School of Immunology & Microbial Sciences, King’s College London, London, UK

## Abstract

Neutralizing monoclonal antibodies (mAbs) targeting severe acute respiratory syndrome coronavirus 2 (SARS-CoV-2) Spike glycoprotein have been developed for the treatment of COVID-19. Whilst antibody therapy has been shown to reduce the risk of COVID-19-associated hospitalization and death, there is limited understanding of the endogenous immunity to SARS-CoV-2 generated in mAb-treated patients and therefore ongoing susceptibility to future infections. Here we measure the endogenous antibody response in SARS-CoV-2-infected individuals treated with REGN-COV2 (Ronapreve). We show that in the majority of unvaccinated, delta-infected REGN-COV2-treated individuals, an endogenous antibody response is generated, but, like untreated, delta-infected individuals, there was a limited neutralization breadth. However, some vaccinated individuals who were seronegative at SARS-CoV-2 infection baseline and some unvaccinated individuals failed to produce an endogenous immune response following infection and REGN-COV2 treatment demonstrating the importance of mAb therapy in some patient populations.

## Introduction

Neutralizing monoclonal antibody (mAb) therapy to treat the symptoms of SARS-CoV-2 has been rapidly developed and licensed [1–3]. The first mAb therapy to be approved for clinical use in the UK was REGN-COV2 (Ronapreve) from Regeneron. This antibody cocktail consists of two receptor-binding domain (RBD) mAbs, REGN10933 (casirivimab) which binds an epitope directly overlapping the ACE2 receptor-binding motif on RBD, and REGN10987 (imdevimab) which binds an epitope on the side of RBD distal to the angiotensin-converting enzyme 2 (ACE2)-binding site [4]. The REGN-COV2 mAb cocktail was shown to protect against mutational escape in preclinical and human studies [5]. Clinical trials Phase I–III also showed that the REGN-COV2 mAb cocktail reduced the viral load, with the greatest effect observed in patients who had not previously had a SARS-CoV-2 infection or vaccination [6]. These trials also showed a reduced risk of COVID-19-associated hospitalization and death [3, 7]. Furthermore, REGN-COV2 treatment in previously uninfected household contacts of infected persons reduced the rate of asymptomatic and symptomatic infections and decreased the duration of symptoms after household exposure [8]. Several studies have reported reduced neutralization potency of REGN-COV2 against omicron sub-lineages including BA.1, BA.2, BA.4 and BA.5 [9–12] and therefore Sotrovimab is the mAb treatment currently being administered in the UK [2].

The aim of this study was to measure the endogenous antibody response generated by the host immune system in patients treated with REGN-COV2 post-SARS-CoV-2 diagnosis to understand ongoing susceptibility to future SARS-CoV-2 infections. The presence of host-derived SARS-CoV-2-specific antibodies was measured using the N-terminal domain (NTD) of Spike, a known target for neutralizing antibodies following natural infection [13, 14]. The presence of endogenous neutralizing activity was measured against a mutated delta Spike where the epitopes for REGN10933 and REGN10987 were disrupted through site-directed mutagenesis, as well as omicron sub-lineages BA.1 and BA.2. We show that whilst endogenous antibody responses can be generated, the breadth of the response is limited in unvaccinated individuals and is similar to untreated individuals. Furthermore, some vaccinated donors who were seronegative at baseline failed to seroconvert post-infection and REGN-COV2 treatment, highlighting the importance of SARS-CoV-2 mAb therapy.

## Materials and methods

### Ethics and cohort description

The collection of surplus serum samples was approved by South Central—Hampshire B REC (20/SC/0310). SARS-CoV-2 cases were diagnosed by reverse transcriptase polymerase chain reaction (RT–PCR) of respiratory samples at St Thomas’ Hospital, London.

### REGN-COV2 treatment criteria and dosage

For patients meeting the treatment criteria (i) a confirmed SARS-CoV-2 infection, (ii) hospitalized for acute COVID-19 and (iii) seronegative for anti-Spike IgG (determined using Abbott SARS-CoV-2 semi-quantitative IgG enzyme immunoassay), a single dose of REGN-COV2 (600 mg casirivimab and 600 mg imdevimab) was administered intravenously.

### COVID-19 severity classification

Disease severity was determined as previously described [17, 18]. Disease severity for patients diagnosed with COVID-19 were classified as follows: (0) asymptomatic or no requirement for supplemental oxygen; (1) requirement for supplemental oxygen [fraction of inspired oxygen (FiO_2_) <0.4] for at least 12 h; (2) requirement for supplemental oxygen (FiO_2_ ≥0.4) for at least 12 h; (3) requirement for non-invasive ventilation/continuous positive airway not a candidate for escalation above level 1 (ward-based) care; (4) requirement for intubation and mechanical ventilation or supplemental oxygen (FiO_2_ >0.8) and peripheral oxygen saturations, 90% [with no history of type 2 respiratory failure (T2RF)] or, 85% (with known T2RF) for at least 12 h; (5) requirement for extracorporeal membrane oxygenation.

### Virus sequencing

SARS-CoV-2 variant infection was confirmed using whole-genome sequencing as previously described [33] or using multiplexed tandem-PCR (MT-PCR) [34].

### Protein expression and purification

Recombinant Spike, N-terminal domain (NTD) and nucleoprotein (N) for enzyme-linked immunosorbent assay (ELISA) were expressed and purified as previously described [17, 35].

### ELISA (Spike and nucleoprotein)

The 96-well plates (Corning, 3690) were coated with antigen at 3 μg/ml overnight at 4°C. The plates were washed (five times with phosphate buffered saline (PBS)/0.05% Tween-20, PBS-T) and blocked with blocking buffer (5% skimmed milk in PBS-T) for 1 h at room temperature. Sera at 1:25 dilution or serial dilutions of sera in blocking buffer were added and incubated for 2 h at room temperature. Plates were washed (five times with PBS-T) and a secondary antibody was added and incubated for 1 h at room temperature. IgG was detected using Goat-anti-human-Fc-AP (alkaline phosphatase) (1:1,000) (Jackson: 109-055-098). Plates were washed (five times with PBS-T) and developed with AP substrate (Sigma) and read at 405 nm.

### Neutralization assay with SARS-CoV-2 pseudotyped virus

Pseudotyped HIV-1 virus incorporating the SARS-CoV-2 Spike protein [either delta (B.1.671.2), delta K444Q/F486K, BA.1 or BA.2] was prepared as previously described [17, 33]. Viral particles were produced in a 10-cm dish seeded the day prior with 5×10^6^ HEK293T/17 cells in 10 ml of complete Dulbecco’s Modified Eagle’s Medium [DMEM-C, 10% foetal bovine serum (FBS) and 1% Pen/Strep] containing 10% (v/v) fetal bovine serum (FBS), 100 IU/ml penicillin and 100 µg/ml streptomycin. Cells were transfected using 90 µg of PEI-Max (1 mg/ml, Polysciences) with: 15µg of HIV-luciferase plasmid, 10 µg of HIV 8.91 gag/pol plasmid and 5 µg of SARS-CoV-2 Spike protein plasmid [36, 37]. The supernatant was harvested 72 h post-transfection. Pseudotyped virus particles were filtered through a 0.45 µm filter and stored at −80°C until required.

Serial dilutions of sera (heat inactivated at 56°C for 30 min) were prepared with DMEM-C media (25µl) (10% FBS and 1% Pen/Strep) and incubated with pseudotyped virus (25 µl) for 1 h at 37°C in half-area 96-well plates. Next, Hela cells stably expressing the ACE2 receptor were added (10 000 cells/25 µl per well) and the plates were left for 72 h. Infection levels were assessed in lysed cells with the Bright-Glo luciferase kit (Promega), using a Victor™ X3 multi-label reader (Perkin Elmer). Each serum sample was run in duplicate and was measured against the five SARS-CoV-2 variants within the same experiment using the same dilution series.

## Results

### Cohort description

Longitudinal serum samples were collected up to 109 days post-treatment from 31 individuals treated with REGN-COV2 at Guy’s and St Thomas’ NHS Foundation Trust between September 2021 and February 2022 (Table 1). At the time of sample collection, the criteria for REGN-COV2 treatment were (i) a confirmed SARS-CoV-2 infection, (ii) hospitalized for acute COVID-19 and (iii) seronegative for anti-Spike IgG by a commercial diagnostic assay (Abbott). Only seven participants had received at least two vaccine doses (either AZD1222 or BNT162b2) of which six were seronegative at SARS-CoV-2 diagnosis. These individuals had underlying health conditions including hypogammaglobulinaemia, acute lymphoblastic leukaemia, thymectomy, chronic lymphocytic leukaemia, peripheral vascular disease, myelodysplasia or had undergone a renal transplant. The remaining 24/31 (77%) participants were unvaccinated and seronegative for Spike prior to antibody administration. Underlying health conditions experienced by unvaccinated participants included asthma, obesity, type-2 diabetes, hypertension, metastatic melanoma, chronic kidney disease, muscular dystrophy and chronic obstructive pulmonary disease. Twenty-eight of the 31 (90%) participants had a confirmed delta infection or were infected in the UK delta wave, and one and two participants had a confirmed alpha and BA.1 infections, respectively. Despite mAb treatment, 16/31 (52%) participants experienced severe disease (disease severity 4 and 5).

**Table 1.** Patient information

Pt	Age	Gender	Underlying health issues	Vaccination status	Last vaccine dose	Previous infection status	Disease severity	Baseline commercial IgG	Date of treatment	Variant
1	36	M	Hypogammaglobulinaemia (X-Linked)	2× AZD1222	5 May21	None known	5	Negative	21 September 2021	Delta
2	24	M	Acute lymphoblastic leukaemia	2× BNT162b2	1 April2021	None known	1	Negative	7 October 2021	Delta
3	39	F	antineutrophil cytoplasmic antibody (ANCA)-associated vasculitis	Unvaccinated	NA	None known	1	Negative	9 October 2021	Delta
4	51	M	Thymectomy	2× AZD1222	20 May 2021	Yes; February2021	0	Negative	8 October 2021	Alpha
5	55	M	Nil	Unvaccinated	NA	None known	5	Negative	20 October 2021	Delta
6	60	M	Nil	Unvaccinated	NA	None known	4	Negative	16 November 2021	Delta
7	53	F	Obese, asthma	Unvaccinated	NA	None known	4	Negative	23 October 2021	Delta
8	74	F	T2DM	Unvaccinated	NA	None known	4	Negative	26 October 2021	Delta
9	53	F	T2DM	Unvaccinated	NA	None known	2	Negative	1 November 2021	Unknown—Presumed Delta
10	79	M	Chronic lymphocytic leukaemia Stage 3, chronic kidney disease, cirrhosis	2× BNT162b2	26 April 2021	None known	0	Negative	4 November 2021	Delta
11	71	M	Renal transplant, T2DM	3× BNT162b2	29 September 2021	None known	4	Negative	6 November 2021	Delta
12	81	F	Hypertension	Unvaccinated	NA	None known	4	Negative	7 November 2021	Delta
13	37	M	Asthma	Unvaccinated	NA	None known	4	Negative	1 November 2021	Delta
14	62	M	Metastatic melanoma	Unvaccinated	NA	None known	4	Negative	11 November 2021	Unknown—Presumed Delta
15	58	M	Metastatic melanoma	Unvaccinated	NA	None known	1	Negative	15 November 2021	Delta
16	81	F	Hypertension	Unvaccinated	NA	None known	4	Negative	18 November 21	Delta
17	27	M	Nil	Unvaccinated	NA	None known	0	Negative	17 November 2021	Delta
18	70	F	Nil	Unvaccinated	NA	None known	4	Negative	21 November 2021	Delta
19	50	F	Chronic kidney disease	Unvaccinated	NA	None known	4	Negative	22 November 2021	Delta
20	44	M	Nil	Unvaccinated	NA	None known	1	Negative	26 November 2021	Delta
21	86	F	Myeloma, asthma	Unvaccinated	NA	None known	4	Negative	29 November 2021	Delta
22	43	F	Muscular dystrophy	Unvaccinated	NA	None known	4	Negative	29 November 2021	Delta
23	69	M	Cirrhosis, chronic obstructive pulmonary disease	Unvaccinated	NA	None known	1	Negative	2 December 2021	Delta
24	46	M	Nil	Unvaccinated	NA	Ever confirmed	4	Negative	3 December 2021	Delta
25	69	F	Hypertension, ischaemic heart disease	Unvaccinated	NA	None known	0	Negative	8 December 2021	Delta
26	78	F	Dementia, T2DM	Unvaccinated	NA	None known	0	Negative	9 December 2021	Delta
27	72	F	T2DM, HTN	Unvaccinated	NA	None known	3	Negative	9 December 2021	Delta
28	63	F	T2DM	Unvaccinated	NA	None known	4	Negative	9 December 2021	Delta
29	96	M	Dementia	Unvaccinated	NA	None known	0	Negative	12 December 2021	Delta
30	70	M	Myelodysplasia, T2DM, chronic obstructive pulmonary disease	2× AZD1222	26 May 2021	None known	2	Not done	13 December 2021	BA.1
31	51	F	Peripheral vascular disease	2× AZD1222	20 May 2021	None known	0	Positive	14 December 2021	BA.1

Information includes: age, gender, underlying health conditions, vaccination status, infecting variant and disease severity (range from 0 to 5). NA - not applicable, M - male, F - female.

### Endogenous antibody responses to SARS-CoV-2 antigens in REGN-COV2 treated patients

**Figure 1.**
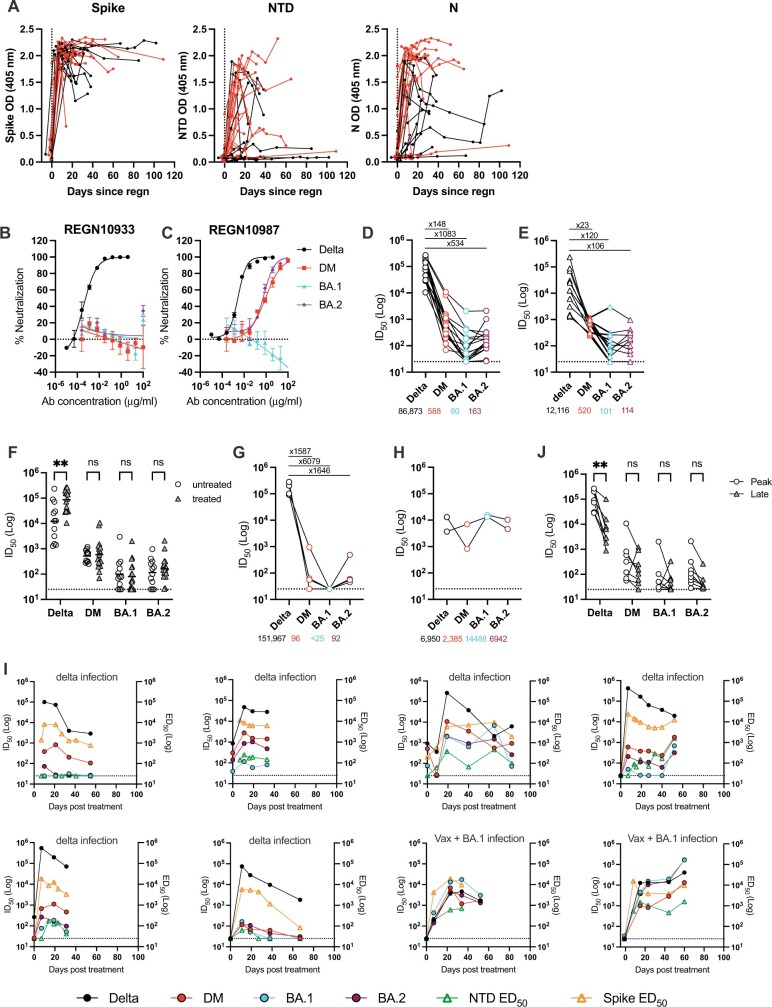
SARS-CoV-2 binding and neutralization activity in sera from REGN-COV2-treated SARS-CoV-2-infected individuals. (**A**) Spike, NTD and N IgG binding in REGN-COV2-treated SARS-CoV-2-infected individuals. Sera was diluted at 1:25 and IgG binding to Spike, NTD and N measured. The vertical dotted line shows the time of REGN-COV2 administration. Participants experiencing severe disease (severity 4 and 5) are shown in red and those experiencing mild disease (severity 0–3) are shown in black. Longitudinal samples from the same individual (*n* = 31) are linked. Neutralization activity was measured against delta variant, delta RBD mutant (DM), BA.1 and BA.2 for (**B**) REGN10933, (**C**) REGN10987, (**D**) sera collected from delta-infected participants (*n* = 17) 16–32 days post-mAb treatment, (**E**) sera collected from unvaccinated, REGN-COV2-untreated, delta-infected individuals (*n* = 12). Horizontal dotted line shows lowest level of detection. GMTs for each virus are shown below the *x*-axis. Fold change in GMT compared to delta shown above. (**F**) Comparison of GMTs between delta-infected individuals who were either treated with REGN-COV2 (*n* = 17, grey triangle) or untreated (*n* = 12, circle). Neutralization activity was measured against delta variant, delta RBD mutant (DM), BA.1 and BA.2 for (**G**) sera collected from vaccinated, REGN-COV2-treated, delta-infected individuals (*n* = 4) and (**H)** sera collected from vaccinated, REGN-COV2-treated, BA.1-infected individuals (*n* = 2). Horizontal dotted line shows lowest level of detection. GMTs are shown below the *x*-axis. Fold change in GMT compared to delta shown above. (**I**) Longitudinal assessment of Spike and NTD ED_50_ for IgG binding, and neutralization activity in REGN-COV2-treated patients. Six individuals were unvaccinated and delta infected, and the remaining two were vaccinated and BA.1 infected as indicated. (**J**) Comparison of ID_50_ between sera collected at 16–23 days (peak) and 55–109 days (late) post-mAb treatment. D’Agostino and Pearson tests were performed to determine normality. Based on this result, multiple Mann–Whitney tests using a two-stage linear step-up procedure of Benjamini, Krieger and Yekutieli were employed to determine significance between groups. ns (non-significant) is *P* > 0.05, *P ≤ 0.05, **P ≤ 0.01, ***P ≤ 0.001 and ****P ≤ 0.0001.

To evaluate host-derived endogenous antibody-binding responses to SARS-CoV-2, we measured the presence of IgG to NTD and N from the Wuhan-1 variant using longitudinal samples as well as binding to recombinant Wuhan Spike by ELISA (Fig. 1A). All baseline samples prior to treatment were seronegative for Spike, N-terminal domain (NTD) and Nucleoprotein (N). As expected, following REGN-COV2 administration, all (31/31) participants showed rapid and high IgG binding to Spike at a 1:25 serum dilution. The presence of IgG binding to NTD and N was delayed in comparison to IgG binding to Spike. Whilst most participants generated Ab responses to NTD and N, 9/31 and 2/31 remained seronegative for NTD and N, respectively, at the time points studied. Four previously vaccinated donors remained seronegative for NTD after SARS-CoV-2 infection.

### Endogenous neutralizing activity in REGN-COV2-treated patients

To measure the endogenous neutralizing activity in sera from REGN-COV2-treated patients, we generated a delta mutant where the binding site of REGN10933 and REGN10987 had been disrupted using K444Q and F486K mutations in RBD (delta RBD mutant, DM) [15, 16]. No detectable neutralization of the delta RBD mutant by REGN10933 was observed at mAb concentrations up to 100 µg/ml (Fig. 1B and C), whereas REGN10987 neutralization was reduced by 328-fold (IC_50_ (antibody concentration that inhibits 50% infection) 0.88 µg/ml). Neutralization was also measured against omicron sub-lineages BA.1 (dominant in the UK from mid-December 2021) and BA.2 (dominant in the UK from February 2022 (Fig. 1B and C). REGN10933 showed no detectable neutralization against BA.2 and neither mAbs were able to neutralize BA.1 at 100 µg/ml. In contrast, the neutralization of BA.2 by REGN10987 was reduced by 197-fold (IC_50_ 0.53 µg/ml).

Neutralization activity was measured in unvaccinated participants with an IgG response to NTD and where a sample was collected 16–32 days post-mAb treatment (*n* = 17). This time window has previously been shown to be the peak of neutralization response following SARS-CoV-2 infection [17]. Due to the administration of REGN-COV2, sera from 17/17 delta-infected individuals collected 16–32 days (median 19 days) post-mAb administration showed very high neutralization titres against delta [geometric mean titre (GMT) 86 873] (Fig. 1D). Neutralization activity against the delta RBD mutant was also detected in 17/17 participants; however, the GMT was reduced by 148-fold (GMT 588) compared to delta, and only one participant had an serum dilution that inhibits 50% infection (ID_50_) <100. Whereas BA.1 was resistant to neutralization by both REGN10987 and REGN10933, serum neutralization activity against BA.1 was detected in 14/17 individuals with a GMT of 80 (1083-fold decrease compared to delta and 7.3-fold compared to delta RBD mutant), indicating a low endogenous heterologous response. BA.2 neutralization was detected in all participants with a GMT of 163 (534-fold reduction compared to delta and 3.4-fold reduction compared to delta RBD mutant). There was no significant difference in the magnitude of the neutralizing activity between those experiencing mild disease (severity 0–3) compared to those experiencing severe disease (severity 4–5) (data not shown).

Serum-neutralizing activity in REGN-COV2-treated participants was compared with untreated individuals who were also unvaccinated and delta-infected 16–22 days post-onset of symptoms [18]. As expected, higher neutralization titres against delta were observed in the treated group compared to the untreated group due to the passive administration of neutralizing REGN-COV2 mAbs (Fig. 1E). However, similar neutralization trends against the delta RBD mutant, BA.1 and BA.2, were observed between the two groups (GMTs 520, 101 and 114, respectively) (Fig. 1F).

Neutralization activity was also measured in six vaccinated individuals experiencing breakthrough infection (four delta and two BA.1 infections) 19–23 days post-mAb treatment (Fig. 1G and H). ID_50_ >100 against the delta RBD mutant and BA.2 were only detected in the donor who had IgG to NTD (Fig. 1G). Four of the four delta-infected individuals failed to produce a BA.1 neutralization response. In contrast, the two participants who had been vaccinated and subsequently BA.1 infected showed robust neutralization against the delta RBD mutant, BA.1 and BA.2 (Fig. 1H).

To investigate the dynamics and longevity of the endogenous antibody response, longitudinal neutralization activity (ID_50_) and half-maximal binding (ED_50_) against Spike and NTD were measured for eight participants with sera collected from baseline up to 82 days post-REGN-COV2 administration (Fig. 1I). For individuals where sera were collected >60 days post-mAb treatment, there was a decline in delta neutralization from the peak as well as a decline in heterologous neutralization (Fig. 1J). NTD and Spike binding mirrored the decrease in neutralization activity over time (Fig. 1I).

## Discussion

Passively acquired antibodies have been shown to inhibit endogenous antibody responses in some situations [19–21]. For example, passively acquired antibodies have been shown to suppress humoral responses in mice immunized with a live attenuated Respiratory Syncytial Virus (RSV) vaccine [19]. Furthermore, maternal antibodies have been shown to suppress infant humoral responses to vaccines against measles [20] and rabies virus [21]. Here we show that in general, the endogenous response to SARS-CoV-2 is not abolished by REGN-COV2 mAb treatment.

A challenge in measuring endogenous antibody responses is the ability to distinguish between the activity of mAb therapy and host-derived immunity. Previous studies have measured endogenous antibody responses by either studying the IgM response to Spike or IgG response to N [22]. Using NTD IgG binding as a measure of the endogenous response, we observed that the majority (20/24) of unvaccinated- and REGN-COV2-treated patients were able to generate an endogenous response that could be detected for >50 days post-mAb treatment. A study of REGN-COV2-treated individuals infected in Wave 1 with the D614G variant similarly showed that the endogenous response to SARS-CoV-2 infection is not abolished but slightly attenuated [23]. We further show that the neutralization breadth of the endogenous response arising from delta infection in unvaccinated individuals was very limited and of a similar level to the untreated control group (Fig. 1F). This observation is also consistent with our previous studies, and those of others, showing that delta infection alone does not generate strong cross-neutralization of the antigenically distant BA.1 and BA.2 in unvaccinated individuals [18, 24, 25]. Reports showing that delta infection poorly protects from BA.1 reinfection [26] suggest that this low level of neutralizing activity is likely insufficient to protect from reinfection with current circulating variants of concern but may be sufficient to prevent severe disease.

Endogenous antibody responses to COVID-19 vaccination following mAb therapy have been shown to have minimal effects on the antibody response in polyclonal sera [27, 28]. However, a recent study by Nussenzweig and colleagues has reported that the presence of high-affinity RBD-specific mAbs can have an impact on the epitopes targeting by vaccine-elicited antibodies [28]. Whether mAb treatment changes epitope immunodominance and B-cell memory in the context of SARS-CoV-2 infection needs to be investigated further.

Of the five vaccinated and delta-infected participants, only one of this group had detectable IgG binding to NTD, suggesting a dysfunctional humoral immune response. A failure to seroconvert following SARS-CoV-2 infection or COVID-19 vaccination has been reported in certain patient populations, including haematological cancer patients [29, 30], haemodialysis patients [31] and solid organ transplant patients [32]. These data highlight the important role of SARS-CoV-2 mAb therapies in treating immunocompromised individuals. In contrast, despite the two BA.1 infected and vaccinated participants being seronegative at baseline, both donors showed robust homologous BA.1 and heterologous delta and BA.2 neutralization activity within 7–8 days post-mAb treatment (Fig. 1H). Although it is not clear whether this response arose from a recall response from prior vaccination and/or a *de novo* response to infection, it demonstrates that passive antibody transfer has not limited the ability to generate serum antibodies with broad neutralization activity.

In summary, we show that many individuals can amount to an endogenous antibody response to SARS-CoV-2 delta infection in the context of REGN-COV2 treatment. However, whether the magnitude and breadth of the neutralization activity are sufficient to protect from reinfection by current SARS-CoV-2 variants of concern and/or severe disease is unknown. These findings provide insights into future susceptibility to SARS-CoV-2 infection in REGN-COV2- or mAb-treated individuals.

## Data Availability

Raw data can be obtained from corresponding author on request.
